# Mitomycin C hypoxic pelvic perfusion for unresectable recurrent rectal cancer: pharmacokinetic comparison of surgical and percutaneous techniques

**DOI:** 10.1007/s13304-017-0480-6

**Published:** 2017-08-08

**Authors:** Stefano Guadagni, Giammaria Fiorentini, Marco Clementi, Paola Palumbo, Andrea Mambrini, Francesco Masedu

**Affiliations:** 1 Section of General Surgery, Department of Applied Clinical Sciences and Biotechnology, University of L’Aquila, via Vetoio, 67100 L’Aquila, Italy; 2 Medical Oncology Unit, Department of Oncology and Hematology, Azienda Ospedaliera “Ospedali Riuniti Marche Nord”, Pesaro, Italy; 3 Department of Life, Health and Environmental Sciences, University of L’Aquila, L’Aquila, Italy; 4 Oncological Unit, Azienda USL Toscana Nord Ovest, Carrara, Massa Carrara Italy

**Keywords:** Unresectable recurrent rectal cancer, Hypoxic pelvic perfusion, Mitomycin C, Stop-flow

## Abstract

**Abstract:**

Patients with unresectable recurrent rectal cancer that progresses after standard and multi-modular treatments are candidates for hypoxic pelvic perfusion. Hypoxic pelvic perfusion can be performed using a surgical or percutaneous approach. The aim of this study was to examine whether the surgical and percutaneous approaches are comparable with respect to tumor drug exposure in the pelvis. A pharmacokinetic study was performed in 18 patients. Both the surgical and percutaneous procedures were performed using mitomycin C (MMC) at a dose of 25 mg/m^2^. The main parameter that was used to evaluate pelvic tumor drug exposure was the ratio of the areas under the MMC plasma concentration curves in the pelvis and the systemic compartment during the perfusion time (AUC_0–20_). The mean values ± SD for the ratios between the MMC AUC_0–20_ in the pelvic and systemic compartments were 14.38 ± 4.31 and 13.15 ± 4.26 for the surgical and percutaneous techniques, respectively (*p* = 0.53). This pharmacokinetic study demonstrated that the percutaneous approach for hypoxic pelvic perfusion did not statistically differ from the surgical approach. When perfusion must be repeated several times in the same patient, the percutaneous and surgical methods may be adopted interchangeably.

**ClinicalTrials.gov Identifier:**

NCT01891552.

## Introduction

The standardization of total mesorectal excision, other refinements in surgical techniques and technologies, and improvements in neoadjuvant and adjuvant therapies have reduced the incidence of locoregional relapse of invasive rectal cancer from approximately 30% of control groups in prospective randomized trials in the 1990s [1, 2] to approximately 6% in the past decade [3]. Management of locally recurrent rectal adenocarcinoma is still a difficult challenge. With standard treatments, the median survival time of patients with unresectable local recurrence of rectal cancer is approximately 1 year [4–9]. When the presence of comorbid conditions contraindicates extensive palliative surgery, when intraoperative irradiation is not available, or when external irradiation is not practical, hypoxic pelvic perfusion has been considered an alternative treatment, as neither intravenous systemic chemotherapy nor intra-arterial chemotherapy can achieve desirable results in terms of pain control and tumor response [10–12].

Several techniques for pelvic perfusion have been proposed [13]. The rationale is to isolate the pelvic circulation by blocking the blood flow in the aorta and the inferior vena cava with balloon catheters and at the level of the thigh with pneumatic cuffs. The pelvis is subsequently perfused with antineoplastic drugs via extracorporeal blood circulation. Unfortunately, the methods that were published were not homogeneous [13]. The simplest and most feasible surgical technique was based on the use of two balloon catheters that were positioned for femoral vessel exposure [10]. With this procedure, a median survival time of 12.2 months was reported at the beginning of the 2000s after one course of mitomycin C (MMC) hypoxic pelvic perfusion in patients with unresectable recurrent rectal cancer that had progressed after chemo-radiotherapy [12].

Improvements have been further explored to identify procedures with enhanced feasibility and reduced morbidity and side effects [14–16]. For repeated perfusion, Thompson et al. proposed a very simple percutaneous hypoxic pelvic perfusion technique that was based on the use of two 18-French (Fr.) introducers [11]; it was later improved by Ricci et al., who used two 11-Fr. introducers [14]. The percutaneous approach with two cannula sheaths was also used by Bonvalot et al. [15] while Begossi et al. [13] and more recently Murata et al. [16] used four cannula sheaths (two 9-Fr. and two 6-Fr.).

The use of a surgical method to expose the femoral vessels was necessary when lymphadenectomy was required, but this method was rarely repeatable because of the resulting scar [13]. A percutaneous approach was more feasible, especially for patients who required procedure repetition. The repetition of perfusion may be useful to prolong clinical responses and survival in a patient with an unresectable relapse of rectal cancer [16].

To examine whether the surgical and percutaneous approaches were comparable in terms of tumor drug exposure in the pelvis, a pilot pharmacokinetic study was performed in 18 patients with unresectable recurrent rectal cancer who had not responded to standard treatments. The drug MMC was used for hypoxic pelvic perfusion, and hemofiltration was added to reduce side effects. MMC was chosen based on previously published studies [12, 17]. Perfusions were performed in hypoxia because MMC is ten times more toxic to tumor cells under hypoxic conditions [18, 19]. Perfusion was performed under normothermic or in mild hyperthermic conditions; the main rationale was to avoid the possibility that mild hypothermia would contribute to immunosuppressive conditions [20]. Outcomes, toxicities, and predictors for outcomes will be published separately.

## Materials and methods

### Eligibility criteria

This study was performed at the University of L’Aquila, L’Aquila, Italy, after approval from the investigational review board and following the consideration that the patients had a disease with a predictable course outcome. Written consent was obtained from 18 patients after they were given complete information about the disease and the implications of the proposed palliative treatment, in accordance with the ethical standards of the committee on human experimentation at our institution. Table 1 shows demographic data. Patients were included in the study if they had unresectable recurrent rectal cancer and had not responded to radiotherapy or systemic chemotherapy, if they had a life expectancy of greater than 3 months, and if they were able to function with some independence (60 on the Karnofsky scale). Subjects with extrapelvic metastases, renal and liver failure, deep venous thrombosis, severe atherosclerosis, or coagulopathy were excluded from this study. This study is a part of a larger study (ClinicalTrials.gov Identifier: NCT01891552). To achieve homogeneity of performance, patients requiring hypoxic pelvic perfusion were referred to our institution.

**Table 1:** Demographic data

Groups	Surgical	Percutaneous
No. of patients	8	10
Gender Male Female	5 3	7 3
Age (years), mean ± SD, [range]	51.89 16.13 [25–72]	67.39 11.47 [49–83]
Weight (Kg), mean ± SD	72.25 ± 5.49	70.05 ± 4.54
Height (cm), mean ± SD	167 ± 8.41	170 ± 10.81
Histology Adenocarcinoma Epidermoid	6 2	9 1
Previous treatments Chemotherapy and radiotherpy Surgery and chemotherapy/radiotherapy	7 1	8 2

*SD* standard deviation

### Hypoxic pelvic perfusion techniques

**Fig. 1:**
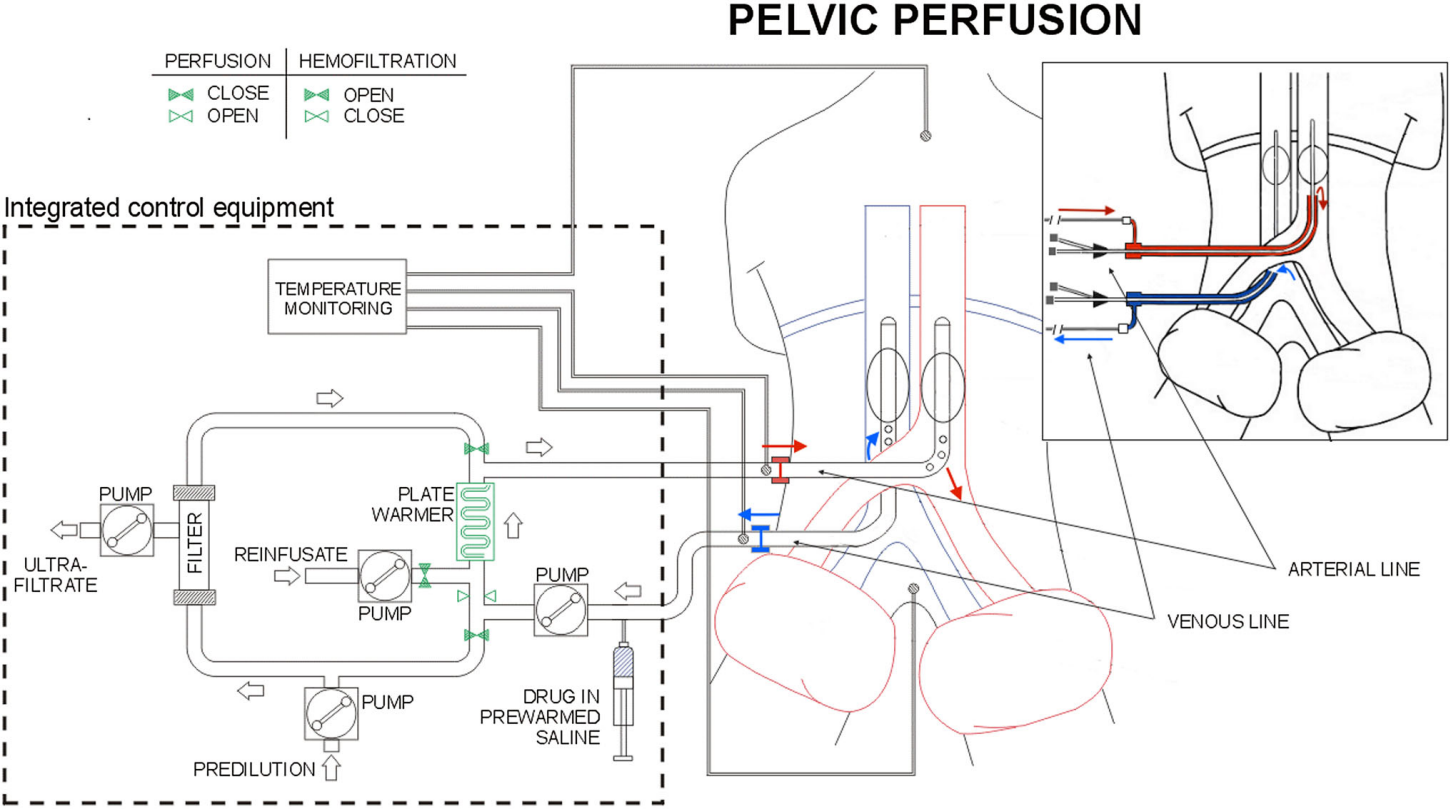
Schema of the surgical and percutaneous (in cartouche) hypoxic pelvic perfusions with hemofiltration

Before undergoing perfusion, patients received an angiography, or angio-CT, of the aorto-iliac tree and the inferior vena cava. Perfusions were performed under general anesthesia, as previously published [12]. Hypoxic pelvic perfusion with hemofiltration has three phases. The first phase is the isolation phase in which the blood flow to the aorta and inferior cava vein is blocked using endovascular balloon catheters and at the level of the thighs using pneumatic cuffs. The isolation phase can be performed by a surgical or percutaneous method (Fig. 1).

**Fig. 2:**
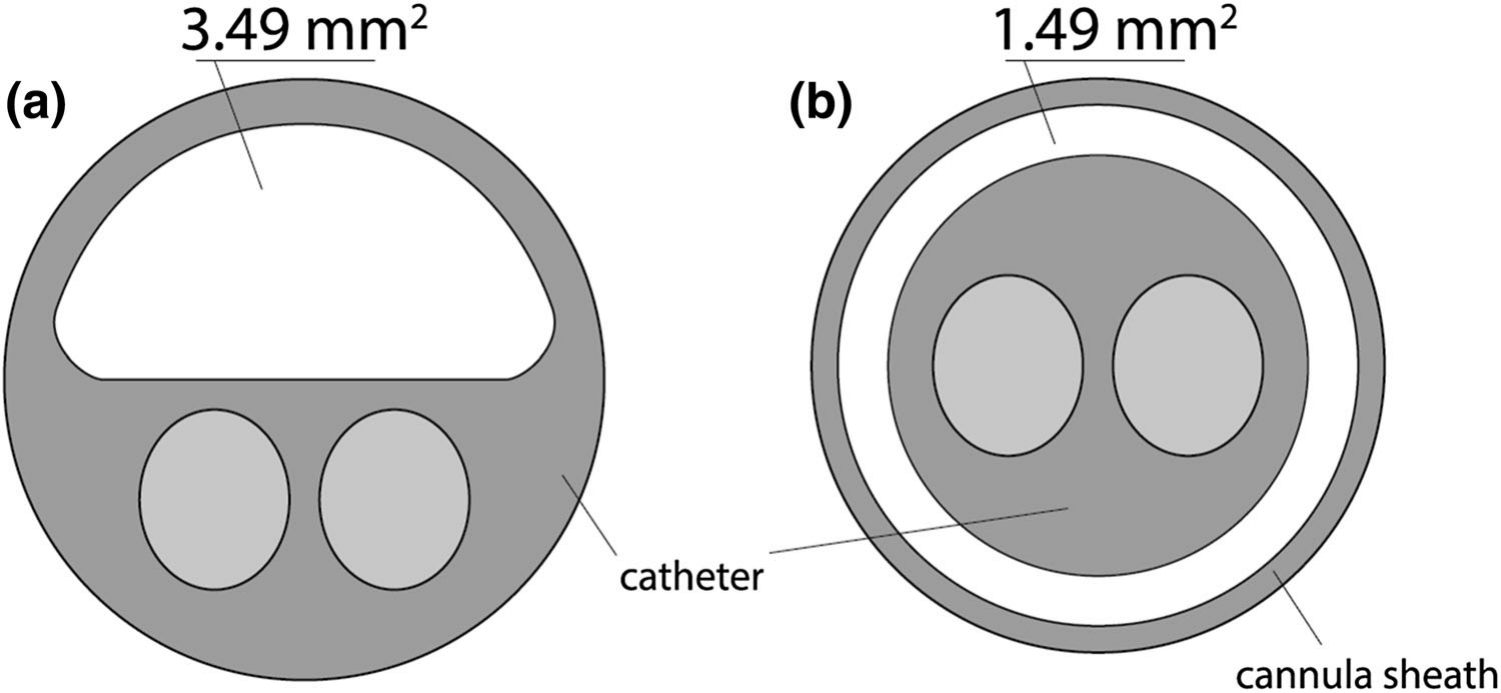
Cross sections; **a** 3-lumen, 12-Fr. balloon catheter (the blood flows in the white area); **b** 2-lumen, 8-Fr. balloon catheter, and 11-Fr. cannula sheath introducer (the blood flows in the white area)

In the surgical approach, after systemic heparinization (150 U/kg heparin), a 3-lumen, 12-Fr. balloon catheter (pfm medical ag, Cologne, Germany) was introduced into the inferior vena cava via the saphenous vein (or iliac vein) and into the aorta via the femoral artery (or iliac artery); the catheters were positioned below the renal vessels and above the aortic and venous bifurcation using a guide wire under fluoroscopic guidance. One of the three lumens of the catheter (Fig. 2a) was used for blood circulation, and the other lumens for inflating the balloons and positioning the guide wire.

The percutaneous procedure requires puncturing the femoral vessels, which was accomplished using two 11-Fr. introducers, each with a hemostatic valve and dilatator (Radifocus; Terumo, Tokyo, Japan). The arterial introducer was 25 cm long and the venous introducer was 10 cm long. We also used two double-lumen 8-Fr. balloon catheters (pfm medical ag). In each catheter, one lumen was used to inflate the balloons and the other lumen was used to position the guide wire. Blood circulation and drug perfusion took place in a long, hollow cylindrical space between the introducer wall and the catheter, with blood flowing through a ring surface at the top of the space (Fig. 2b).

To suspend the blood flow, the balloons were inflated with the radiopaque dye diatrizoate diluted in an isotonic sodium chloride solution. To complete the isolation of the pelvis, two large-cuff orthopedic tourniquets were placed around each of the thighs and inflated just before starting the perfusion.

The second phase is the perfusion phase in which perfusion occurs via extracorporeal blood circulation. The infusion channels of the arterial and venous surgical catheters or the hemostatic valve channels of the 11-Fr. introducers were connected to a hypoxic perfusion tubing set mounted on a regional cancer therapy-dedicated circulation device (Performer LRT; RanD, Medolla, Italy). The circuit was primed with an isotonic sodium chloride solution containing heparin (10,000 U/L). Once the requisite blood flow (approximately 100 mL/min) in the extracorporeal circuit (aspiration from the inferior cava vein and infusion into the aorta) was established, drug perfusion was started.

MMC (25 mg/m^2^), diluted in 250 mL of an isotonic sodium chloride solution containing 16 mg of dexamethasone sodium phosphate, was administered via the circuit over a 3-min period. The extracorporeal circuit (Fig. 1) connected to the circulation device contains a heating element and a hemofiltration module, both of which are controlled by the device during the perfusion phase and subsequent hemofiltration phase. Perfusion was administered for 20 min. The temperature loss was approximately 1 °C per meter of the tubing set. The length of the tubing set was 5 m; hence, the temperature required to ensure normothermia was 42 °C at the outlet level of the heating element. The circulation device has sensors that monitor and regulate the blood flow, withdrawal pressure, infusion pressure, circuit and patient temperatures, hemofiltration parameters, and save the data.

The third phase is the hemofiltration phase. After perfusion, the catheter balloons and pneumatic cuffs were deflated and normal circulation was restored. Hemofiltration was then administered for 60 min via the circuit. The blood flow was increased to approximately 200 mL/min and, to maintain this flow, the aorta was the withdrawal site. The temperature at the outlet level of the heating element was decreased to 39 °C. A polyamide hemofilter with a surface area of 2.1 m^2^ (RanD, Medolla, Italy) was used for filtration. After filtration, the catheters were withdrawn and the vessels were repaired. Approximately 30 min of compression hemostasis was administered after percutaneous technique. Protamine was injected at 200 IU/kg to reverse the anticoagulant effects of heparin.

### MMC regimen and pharmacokinetic study

MMC was administered at a dose of 25 mg/m^2^, in accord with previously published studies [12, 17]. Hemofiltration was performed in all procedures.

The pharmacokinetic study was conducted with eight patients who underwent the surgical procedure and ten patients who underwent the percutaneous method. For sample collection and analysis, pelvic (inferior vena cava) and systemic (peripheral vein of the arm) blood samples were obtained during the 20-min perfusion period at 5, 7, 14, 17, and 20 min. At the end of the perfusion and after balloon deflation, additional samples were obtained from the systemic blood, from blood that was exiting the patient and passing through the extracorporeal circuit, and from the ultrafiltrate at 25, 30, 45, 60, and 120 min. All samples (2 mL in heparinized tube) were immediately processed for storage. The blood samples were centrifuged at 3000*g* for 10 min and the plasma from the blood samples was transferred to capped polypropylene tubes for storage. All samples were stored at −20 °C until high-performance liquid chromatography (HPLC) analysis of the MMC concentrations [21]. HPLC analysis for all patient samples was performed within 24 h of collection. For the pharmacokinetic study, a non-compartment pharmacokinetic analysis was fitted to the MMC concentration–time data [22]. For the hemofiltration from 20 to 120 min, the total MMC removal (TMMCR) was calculated from the ratio between the MMC concentrations in the ultrafiltrate and the MMC concentrations in blood exiting the patient. The pH and pO_2_ were also measured in the blood samples that were collected from the extracorporeal circuit at 5, 7, 14, 17, and 20 min during the isolated perfusion phase.

### Statistical analysis

The analysis provided descriptive statistics, means, and standard deviations (SDs). The comparisons between the surgical and percutaneous treatments with respect to pharmacokinetic, biochemical, and hemodynamic parameters were performed using non-parametric Mann–Whitney tests. If no statistically significant difference emerged in the comparison, multiple testing adjustment was not required and type I error was set at 0.05.

The statistical analysis was performed using STATA software, version 14 (StataCorp, College Station, Texas).

## Results

**Fig. 3:**
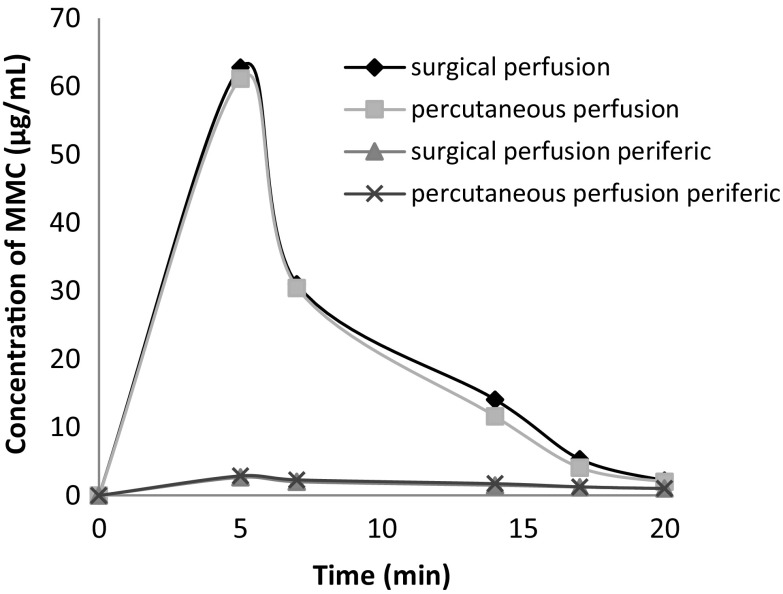
MMC concentrations in blood from inferior vena cava (0–20 min) and peripheral blood (0–20 min) for the surgical and percutaneous groups

**Fig. 4:**
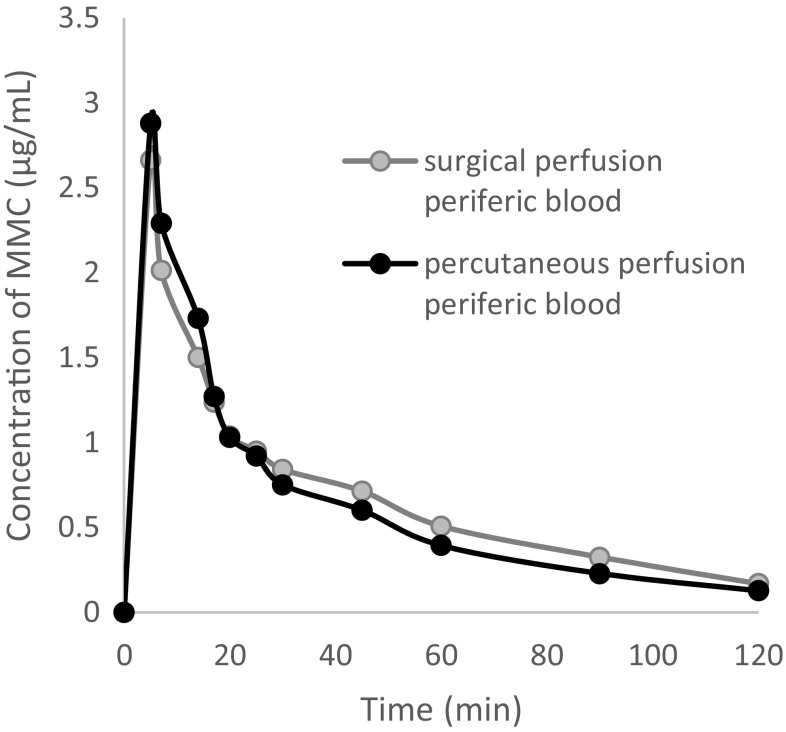
MMC concentrations in peripheral blood (0–120 min) for the surgical and percutaneous groups

Figure 3 shows MMC concentrations in blood from inferior vena cava (0–20 min) and in peripheral blood (0–20 min) for surgical technique and percutaneous technique groups. Figure 4 shows MMC concentrations in peripheral blood (0–120 min) for the surgical technique and percutaneous technique groups.

Table 2 reports the mean values ± SD of blood flow, withdrawal pressure, infusion pressure, rectal temperature, esophageal temperature, extracorporeal blood pH, and pO_2_. All these parameters were recorded every 60 s by the dedicated apparatus during the 20-min perfusion. Data from eight patients who received IPP via a surgical technique were compared to data from ten patients who were treated with the percutaneous method and the two techniques did not show statistically significant differences. Table 2 in particular shows that the mean values for blood flow did not significantly differ between the two groups (approximately 100–120 mL/min), which confirmed that both procedures were performed under similar conditions.

**Table 2:** Mean values ± standard deviation (SD) of blood flow, withdrawal pressure, infusion pressure, rectal temperature, esophageal temperature, extracorporeal blood pH, and pO_2_ recorded during surgical (eight patients) versus percutaneous (ten patients) hypoxic pelvic perfusions

Technique	Surgical		Percutaneous		MW
	Mean ± SD	Range	Mean ± SD	Range	*p* value (na)
Blood flow (ml/min)	117.06 ± 17.00	84.5/137.75	122.50 ± 24.32	100/164.75	0.98 (ns)
Withdrawal pressure (mmHg)	−13.49 ± 23.51	−34.7/23.8	−26.68 ± 11.47	−37/−5.9	0.07 (ns)
Infusion pressure (mmHg)	102.56 ± 8.89	92.4/113.1	115.22 ± 18.03	90.1/143.1	0.11 (ns)
Rectal temperature (°C)	37.30 ± 0.66	36.32/38.39	37.43 ± 0.29	37.01/37.8	0.59 (ns)
Esophageal temperature (°C)	35.91 ± 0.48	35.22/36.76	36.18 ± 0.32	35.73/36.54	0.21 (ns)
pH extracorporeal circuit	7.38 ± 0.02	7.38 ± 0.02	7.37 ± 0.02	7.34/7.4	0.42 (ns)
pO_2_ extracorporeal circuit (mmHg)	28.52 ± 0.64	27.8/29.5	28.66 ± 0.55	27.9/29.7	0.56 (ns)

The techniques were compared during the hypoxic perfusion time (20 min)

*MW* Mann–Whitney test, *ns* not significant, *na* not adjusted for multiple testing

Table 3 reports mean values ± SD for the ratio between the areas under the MMC plasma concentration curves (AUC_0–20_) in the pelvic and systemic compartments, for the maximal MMC plasma concentration (*C*_max_), and for the ratio of the MMC *C*_max_ values in the pelvic and systemic compartments. Among these pharmacokinetic tested parameters, the two techniques did not show statistically significant differences.

**Table 3:** Pharmacokinetic and biochemical characteristics of 25 mg/m^2^ MMC during surgical (eight patients) versus percutaneous (ten patients) hypoxic pelvic perfusions

Technique	Surgical		Percutaneous		MW
	Mean ± SD	Range	Mean ± SD	Range	*p* value (na)
AUC_0–20_ perfused compartment/AUC_0–20_ peripheric compartment	14.38 ± 4.31	8.79/20.83	13.15 ± 4.26	7.37/19.21	0.53 (ns)
*C*_max_ perfused compartment (µg/ml)	62.76 ± 14.75	40.1/79.2	61.12 ± 11.84	46.8/79.3	0.72 (ns)
*C*_max_ perfused compartment/*C*_max_ peripheric compartment	23.77 ± 8.18	14.77/38.41	22.19 ± 5.99	14.96/33.41	0.72 (ns)

The techniques were compared during the hypoxic perfusion time (20 min)

*MW* Mann–Whitney test, *ns* not significant, *na* not adjusted for multiple testing, *AUC*_*0–20*_ area under the plasma concentration curve (0–20 min), *C*_*max*_ maximum plasma concentration

Table 4 reports other pharmacokinetic parameters in the peripheral blood that were calculated during the entire procedure time (0–120 min). Mean values ± SD of volume of distribution (*V*_d_), half-time of elimination (*t*_½_), clearance (Cl), and TMMCR (during the hemofiltration phase 20–120 min) are presented. Among these pharmacokinetic tested parameters, the two techniques did not show statistically significant differences.

**Table 4:** Pharmacokinetics of 25 mg/m^2^ MMC in the peripheral venous blood during surgical (eight patients) versus percutaneous (ten patients) hypoxic pelvic perfusions

Technique	Surgical		Percutaneous		MW
	Mean ± SD	Range	Mean ± SD	Range	*p* value (na)
*C*_max_ (µg/ml)	2.66 ± 0.78	1.7/3.9	2.88 ± 0.75	1.9/4.2	0.62 (ns)
AUC_0–120_ (µg/ml min)	81.79 ± 19.01	58.95/106.7	74.86 ± 19.21	56.57/114.55	0.53 (ns)
*t*_1/2_ (min)	39.25 ± 7.14	30.03/50.32	34.63 ± 3.72	26.96/38.9	0.15 (ns)
*V*_d_ [mg/(µg/ml)]	26.08 ± 8.08	15.77/37.88	25.90 ± 6.24	17.10/33.33	0.92 (ns)
Cl [mg/(µg/ml)/min]	0.46 ± 0.10	0.33/0.61	0.51 ± 0.12	0.31/0.66	0.27 (ns)
TMMCR (%)	28.61 ± 2.72	23.72/32.97	31.03 ± 2.53	28.25/37.11	0.32 (ns)

The techniques were compared during perfusion (0–20 min) and hemofiltration (20–120 min) phases; for TMMCR, comparison was made during the hemofiltration phase (20–120 min)

*MW* Mann–Whitney test, *ns* not significant, *na* not adjusted for multiple testing, *AUC*_*0–120*_ area under the plasma concentration curve (0–120 min), *t*_*1/2*_ half-life of elimination phase, *V*_*d*_ volume of distribution, *Cl* total clearance (extracorporeal plus systemic), *TMMCR* total MMC removal

## Discussion

Approximately 10–18 months of survival have been reported after a single course of hypoxic pelvic perfusion in patients with unresectable recurrent rectal cancer that had progressed following radiotherapy or systemic chemotherapy [12, 23–27]. When hypoxic pelvic perfusion was routinely performed twice, a mean survival time of 24 months was reported [16], supporting the hypothesis that repeated perfusion may be useful to prolong clinical responses and survival.

Hypoxic pelvic perfusion can be performed using a surgical or a percutaneous approach. This study was performed to assess whether the two approaches were comparable in terms of pelvic drug concentration and whether they could thus be used interchangeably in a single patient. The MMC concentration was analyzed in venous blood from the pelvis and in systemic blood to study the differences between pelvic and systemic drug bioavailability during the perfusion phase with vascular blockage. A further analysis of the MMC concentration in the systemic blood and in the blood ultrafiltrate was performed to assess how much drug was eliminated during hemofiltration.

Both the surgical and percutaneous procedures were performed using MMC at a dose of 25 mg/m^2^ in a homogeneous group of 18 patients with unresectable recurrent rectal cancer who had not responded to standard treatments. Sample size is a little limitation and does not attenuate the statistical power of the analysis. A reliable comparison between the surgical and percutaneous approaches could be made only if the perfusion phase with extracorporeal blood circulation was exactly the same, particularly with respect to blood flow. The percutaneous approach required a higher withdrawal pressure relative to the surgical approach, but the difference was not statistically significant, and the use of dedicated equipment guaranteed the same mean blood flow rate in the extracorporeal circuit. The most substantial observation in this study is that the MMC bioavailability in the perfused pelvic compartment did not differ between the two groups, as demonstrated by the mean values of the MMC AUC_0–20_ ratios.

The rationale of 20 min perfusion time was based on in vitro chemosensitivity studies [28], in vitro cellular uptake studies [29], and in vivo pharmacokinetics studies [17].

**Fig. 5:**
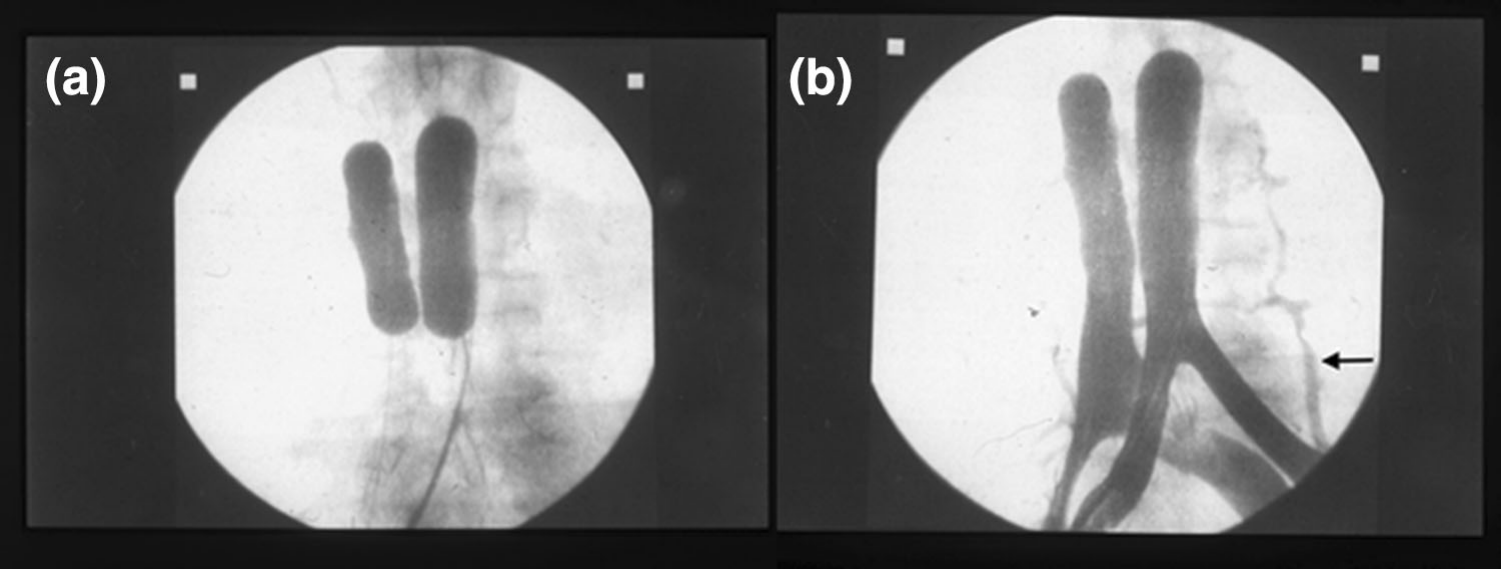
**a** Hypoxic pelvic perfusion: endovascular occlusion of the inferior vena cava and the aorta with blood flow blockade at the level of the thighs; **b** Hypoxic pelvic perfusion: after contrast injection, the pelvic compartment was defined, and leakage through the retroperitoneal vessels (*arrow*) was detected

Slow-flow perfusion was adopted to reduce leakage and, therefore, to lower systemic toxicity because of a well-documented relationship [13] between the flow in the circuit and drug leakage to the systemic circulation. Figure 5a shows an endovascular occlusion of the inferior vena cava and the aorta with blood flow blockage at the level of the thighs. After contrast injection, the pelvic compartment was defined and leakage through retroperitoneal vessels was detected (Fig. 5b). Pelvic perfusion was immediately followed by hemofiltration to reduce systemic side effects. Hemofiltration has been preferred over other methods as a protective measure against toxicity [15, 30]. The average TMMCR in the venous blood during the hemofiltration phase (20–120 min) was approximately 30%.

## Conclusions

In terms of tumor drug exposure, this MMC pharmacokinetic study of patients with unresectable recurrent rectal cancer demonstrated that the percutaneous approach for hypoxic pelvic perfusion was not significantly different from surgical approach. When perfusion must be repeated several times in the same patient, the percutaneous and surgical methods may be interchangeably adopted without a loss in clinical homogeneity or therapeutic efficacy, provided that the perfusion-phase parameters, particularly the blood flow rate, are consistent.
